# Supplementary material for: Lockdowns, lethality, and laissez-faire politics. Public discourses on political authorities in high-trust countries during the COVID-19 pandemic
Source: PLoS One. 2021 Jun 23;16(6):e0253175. doi: 10.1371/journal.pone.0253175 (PMC8221506; doi:10.1371/journal.pone.0253175)
Supplement: S1 Appendix — Includes Tables A through D. (PDF) [file pone.0253175.s001.pdf]

S1 Appendix: Timelines (by country)

Table A: COVID-19 timeline, Denmark	
February 27th	▪ First Danish case of COVID-19
February 28th	▪ Danish Health Authority holds first press briefing on the status of COVID-19 in Denmark.
March 6th	▪ Prime Minister Mette Frederiksen holds 1st press conference, recommends that all events with more than 1000 participants be canceled. It is recommended to limit physical contact. The Foreign Ministry changes all green zones to yellow zones.
March 10th	▪ Prime Minister holds 2nd press conference. The Foreign Ministry cautions against traveling to several regions in Italy, Iran, China, South Korea, and Austria. Flights to these areas stopped.<br>▪ A new bailout makes it possible for companies to seek compensation for canceled events with more than 1000 participants.
March 11th	▪ Prime Minister holds 3rd press conference: Denmark will go on lockdown for the next two weeks. All non-essential public personnel is sent home. Schools, childcare facilities, and institutions close. A ban on gatherings of more than 100 people is implemented.
March 12th	▪ Business bailout passed.
March 13th	▪ Prime Minister holds 4th press conference. Borders will be closed the next day. The foreign ministry advises against all unnecessary travel, and all Danes travelling abroad are advised to return home.
March 14th	▪ Denmark closest borders as one of the first countries in the world.<br>▪ The first COVID-infected Dane dies. Non-acute medical treatments are postponed to free up capacity in the health care sector.<br>▪ The government grants 10 million kroner to COVID-19 research.
March 15th	▪ Prime Minister holds 5th press conference. A tripartite agreement has been established, ensuring financial support for larger companies that have had to send home employees.
March 17th	▪ Prime Minister holds 6th press conference. Gathering ban tightened to 10 people from the next day.<br>▪ The Queen addresses the nation
March 20th	▪ 19 Danes have died from COVID-19
March 21st	▪ government grants 100 million kroner in financial aid to help developing countries fight Covid-19
March 23rd	▪ Prime Minister holds 7th press conference. All containment measures extended until April 13th.
March 30th	▪ Prime Minister holds 8th press conference. If the positive development continues, Denmark may slowly reopen after Easter.
March 31st	▪ 109 Danes have died from COVID-19
April 6th	▪ Ban on large gatherings extended until August<br>▪ Prime Minister holds 9th press conference, presents reopening plan. Childcare institutions and Primary School reopen from mid-April.
April 10th	▪ 260 Danes have died from COVID-19
April 14th	▪ Prime Minister holds 10th press conference. Development so positive Denmark may be reopened to a further degree than originally planned.
April 16th	▪ 336 Danes have died from COVID-19
April 17th	▪ Some private-sector industries to open from April 20th.
April 18th	▪ Government adjusts and expands business bailout.<br>▪ Some non-acute medical treatments may be performed again.
April 21st	▪ Increased testing<br>▪ Government passes ban on the gatherings of more than 500 people until September 1st.
April 23rd	▪ 394 Danes have died from COVID-19

TABLE B: COVID-19 TIMELINE, GERMANY	
January 27th	▪ First German case of COVID-19. German Health Authority does not expect further spread.
February 12th	▪ Minister of Health Jens Spahn presents containment strategy in Parliament
February 25th	▪ Germany and neighboring countries agree that border control and cancellation of large events will be ineffective
February 26th	▪ New cases in Nordrhein-Westfalen
February 28th	▪ Germany increases test capacity
March 2nd	▪ Minister of Health holds press conference, presents recommendations on protective behavior<br>▪ Germany donates financial aid to help Iran combat COVID-19<br>▪ German Health Authority increases risk level of COVID-19 to "moderate"
March 3rd	▪ Germany introduces export ban on healthcare equipment
March 9th	▪ First German dies from COVID-19
March 10th	▪ Minister of Health recommends that all events with more than 1000 participants be canceled
March 11th	▪ Chancellor Angela Merkel addresses the nation for the first time
March 12th	▪ Chancellor encourages the public to practice social distancing
March 13th	▪ All schools and kindergartens close
March 15th	▪ Germany goes into lockdown, closing borders as well as places of social gathering, e.g. gyms, restaurants, and churches
March 17th	▪ Minister of health encourages public to show solidarity and practice social distancing<br>▪ German health authority increases risk level of COVID-19 to "high". 24 Germans have died from COVID-19
March 20th	▪ 52 Germans have died from COVID-19
March 22nd	▪ contact ban introduced, prohibits gatherings of more than two people
March 26th	▪ Germany to take 47 COVID-19 patients from Italy
April 1st	▪ 821 Germans have died from COVID-19
April 9th	▪ Minister of Health encourages public to practice social distancing over Easter
April 10th	▪ 2706 Germans have died from COVID-19
April 15th	▪ contact ban extended until May 3rd
April 20th	▪ 4642 Germans have died from COVID-19

Table C: COVID-19 timeline, The Netherlands	
February 27th	▪ First Dutch case of COVID-19
March 5th	▪ 82 confirmed cases in the Netherlands
March 6th	▪ First Dutchman has died from COVID-19
	▪ Special containment measures for Noord-Brabant introduced
March 9th	▪ Minister-President Mark Rutte encourages population to avoid shaking hands
March 12th	▪ Events with more than 100 participants are canceled, people encouraged to work from home if possible.
March 15th	▪ Schools and childcare institutions close, places of social gathering, e.g. restaurants and sports clubs close.
March 17th	▪ Business bailout passed.
March 19th	▪ Minister of health Bruno Bruins quits due to burnout
March 20th	▪ Test kit shortage: testing requirements tightened
	▪ 106 Dutchmen have died from COVID-19
March 23rd	▪ new containment measures: Cancelation of events extended to June 1, municipalities given authority to close stores and close off crowded areas
March 26th	▪ Government bans groups in public
March 31st	▪ Containment measures extended until April 28th
April 1st	▪ 1173 Dutchmen have died from COVID-19
April 5th	▪ Number of intensive care beds doubled
April 6th	▪ Test capacity increased
April 8th	▪ Pressure on intensive care units eases up
April 10th	▪ 2511 Dutchmen died from COVID-19
April 15th	▪ Minister-President discusses prerequisites for reopening.
April 20th	▪ 3751 Dutchmen have died from COVID-19
April 21st	▪ The government announces that primary schools are to reopen on May 11th, the ban on public events is extended to September 1st

Table D: COVID-19 timeline, Sweden	
January 31st	▪ First Swedish case of COVID-19 (no further contagion)
February 27th	▪ COVID-19 reintroduced to Sweden.
March 2nd	▪ COVID-19 risk of new cases in Sweden adjusted to "very high", risk for community spread adjusted to "moderate".
March 6th	▪ Swedish Health Authority (Folkhälsomyndigheten) recommends testing people returning from affected areas in Northern Italy.
March 11th	▪ Government holds extraordinary press conference, bans gatherings of more than 500 participants
March 16th	▪ Government passes emergency bailout
March 18th	▪ Government encourages universities and high schools to switch to online learning
March 19th	▪ Swedish Health Authority encourages public to consider avoiding traveling if unnecessary, implements a travel ban for non-EU citizens
March 20th	▪ 30 Swedes that died from COVID-19
March 24th	▪ Restaurants, cafes and bars subject to increased regulation
March 29th	▪ Ban on gatherings of more than 50 people
April 1st	▪ National visitation ban on retirement homes implemented<br>▪ 385 Swedes have died from COVID-19
April 9th	▪ Government addresses criticism of being intransparent
April 10th	▪ 1155 Swedes have died from COVID-19
April 20th	▪ 2097 Swedes have died from COVID-19

References (S1 Appendix)

1. Bundesministerium für Gesundheit. Coronavirus SARS-CoV-2: Chronik der bisherigen Maßnahmen. Bundesministerium für Gesundheit, 2020 Aug 06 [cited 2020 Aug 14]. German. Available from: https://www.bundesgesundheitsministerium.de/coronavirus/chronik-coronavirus.html
2. Dansk Erhverv. 2020 Mar 18 [cited 2020 Aug 14]. Forbud mod arrangementer -hvad gælder? Danish. Available from: https://www.danskerhverv.dk/corona/forsamlingsforbud/
3. Darroch, G. Coronavirus: A timeline of the pandemic in the Netherlands. Dutch News. 2020 May 16 [cited 2020 Aug 14]. Available from https://www.dutchnews.nl/news/2020/05/coronavirus-a-timeline-of-the-pandemic-in-the-netherlands/
4. Folkhälsomyndigheten. Nya regler för restauranger och krogar. Folkhälsomyndigheten. 2020 March 24 [cited 2020 Aug 14]. Swedish. Available from https://www.folkhalsomyndigheten.se/nyheter-och-press/nyhetsarkiv/2020/mars/nya-regler-for-restauranger-och-krogar/
5. Justitiedepartementet. Frågor och svar – tillfälligt inreseförbud till EU via Sverige. Regeringskansliet. 2020 May 15 [cited 2020 Aug 14]. Swedish. Available from: https://www.regeringen.se/artiklar/2020/04/fragor-och-svar-inresestopp-till-eu-via-sverige/
6. Krisinformation.se. Restriktioner och förbud. Krisinformation.se. 2020 May 12 [cited 2020 Aug 14]. Swedish. Available from: https://www.krisinformation.se/detta-kan-handa/handelser-och-storningar/20192/myndigheterna-om-det-nya-coronaviruset/restriktioner-och-forbud

7. nu.nl. Tijdlijn: Het coronavirus in Nederland. nu.nl. 2020 Apr 07 [cited 2020 Aug 14]. Dutch. Available from: https://www.nu.nl/coronavirus/6040831/tijdlijn-het-coronavirus-in-nederland.html

8. Ohlin, E. Sveriges andra coronafall bekräftat. Läkartidningen. 2020 Feb 27 [cited 2020 Aug 14]. Swedish. Available from: https://lakartidningen.se/aktuellt/nyheter/2020/02/sveriges-andra-coronafall-bekraftat/

9. Radtke, R. Erkrankungs- und Todesfälle mit Coronavirus (COVID-19) in Deutschland 2020. 2020 Aug 14 [cited 2020 Aug 14]. In: Statista [internet]; [1 screen]. German. Available from: https://de.statista.com/statistik/daten/studie/1102667/umfrage/erkrankungs-und-todesfaelle-aufgrund-des-coronavirus-in-deutschland/

10. Resumé. Tidslinje: Så har coronakrisen slagit mot branschen – dag för dag. Resumé. 2020 Apr 28 [cited 2020 Aug 14]. Swedish. Available from: https://www.resume.se/insikt/guide/tidslinje-sa-har-coronakrisen-slagit-mot-branschen-dag-for-dag/

11. Rønnstad, E. B. TIDSLINJE OVER CORONAKRISEN: HVAD SKETE DER OG HVORNÅR? Tænketanken Europa. 2020 May 04 [cited 2020 Aug 14]. Danish. Available from: http://thinkeuropa.dk/politik/tidslinje-over-coronakrisen-hvad-skete-der-og-hvornaar

12. Statsministeriet. Pressemøde 17. marts 2020 om yderligere tiltag mod coronavirus [video; press release]. In: stm.dk [internet]. 2020 Mar 17 [cited 2020 Aug 14]. Danish. Available from: http://video.stm.dk/pressemode-17-marts-2020-om

13. SVT Nyheter. Första coronafallet i Sverige. SVT Nyheter. 2020 Jan 31 [cited 2020 Aug 14]. Swedish. Available from: https://www.svt.se/nyheter/snabbkollen/forsta-virusfallet-i-sverige

14. SVT Nyheter. Gymnasieskolor och högskolor rekommenderas distansundervisning. SVT Nyheter. 2020 Mar 17 [cited 2020 Aug 14]. Swedish. Available from:

https://www.svt.se/nyheter/inrikes/gymnasieskolor-och-hogskolor-rekommenderas-distansundervisning

15. SVT Nyheter. Lista: Här är restriktionerna som gäller i Sverige. SVT Nyheter. 2020 Mar 25 [cited 2020 Aug 14]. Swedish. Available from: https://www.svt.se/nyheter/inrikes/lista-har-ar-restriktionerna-som-galler-i-sverige

16. SVT Nyheter. Så har regeringen agerat mot corona-pandemin. SVT Nyheter. 2020 Apr 03 [cited 2020 Aug 14]. Swedish. Available from: https://www.svt.se/nyheter/inrikes/sa-har-regeringen-agerat-mot-corona

17. Tagesschau. Einigung auf umfassendes Kontaktverbot. Tagesschau. 2020 Mar 22 [cited 2020 Aug 14]. German. Available from: https://www.tagesschau.de/inland/kontaktverbot-coronavirus-101.html

18. Welt. Maßnahmen, die es so in unserem Land noch nicht gegeben hat. Welt. 2020 Mar 16 [cited 2020 Aug 14]. German. Available from: https://www.welt.de/politik/deutschland/article206590517/Coronavirus-Was-in-Deutschland-jetzt-schliessen-soll-und-was-nicht.html

19. World-o-meters. (2020). Coronavirus: Sweden. 2020 Aug 13 [cited 2020 Aug 13]. In: World-o-meters [internet]; [4 screens]. Available from: https://www.worldometers.info/coronavirus/country/sweden/
