# Supplementary material for: Lockdowns, lethality, and laissez-faire politics. Public discourses on political authorities in high-trust countries during the COVID-19 pandemic
Source: PLoS One. 2021 Jun 23;16(6):e0253175. doi: 10.1371/journal.pone.0253175 (PMC8221506; doi:10.1371/journal.pone.0253175)
Supplement: S2 Appendix — Includes a single table, Table A. (PDF) [file pone.0253175.s002.pdf]

S2 Appendix: Twitter search terms

TABLE A: TWITTER SEARCH TERMS BY COUNTRY AND THEME

Term set	Base set (authority terms + language)	COVID-19 search terms
Denmark	("Mette Frederiksen" OR statsminister OR statsministeren OR regering OR regeringen OR @RegeringDK) lang:da	(corona OR coronavirus OR covid19 OR covid-19 OR covid_19 OR #corona OR #coronavirus OR #coronacrisis OR #corona2020 OR #covid19 OR #covid-19 OR #covid_19 OR #covid19dk OR #covid19danmark OR #coronadk OR #coronadanmark OR #coronavirusdk OR #coronavirusdanmark)
Germany	(Merkel OR Bundeskanzlerin OR Kanzlerin OR Regierung OR @AngelaMerkelCDU) -Österreich -österreichisch -österreichische -Bierlein -Bundeskanzler -ÖVP -Schweiz -schweizisch -schweizische -Sommaruga -Bundespräsidentin lang:de	(corona OR coronavirus OR covid19 OR covid-19 OR covid_19 OR #corona OR #coronavirus OR #coronacrisis OR #corona2020 OR #covid19 OR #covid-19 OR #covid_19 OR #covid19de OR #covid19deutschland OR #coronade OR #coronadeutschland OR #coronavirusde OR #coronavirusdeutschland)
The Netherlands	(Rutte OR minister-president OR regering OR @MinPres) -Vlaanderen -vlaams -vlaamse -Jambon -Homans -Vervoort -België -belgisch -Brussels lang:nl	(corona OR coronavirus OR covid19 OR covid-19 OR covid_19 OR #corona OR #coronavirus OR #coronacrisis OR #corona2020 OR #covid19 OR #covid-19 OR #covid_19 OR #covid19nl OR #covid19nederland OR #coronanl OR #coronanederland OR #coronavirusnl OR #coronavirusnederland)
Sweden	("Stefan Löfven" OR statsminister OR statsministern OR regering OR regeringen OR @SwedishPM) lang:sv	(corona OR coronavirus OR covid19 OR covid-19 OR covid_19 OR #corona OR #coronavirus OR #coronacrisis OR #corona2020 OR #covid19 OR #covid-19 OR #covid_19 OR #covid19sv OR #covid19sverige OR #coronasv OR #coronasverige OR #coronavirussv OR #coronavirussverige)
