# Supplementary material for: Lockdowns, lethality, and laissez-faire politics. Public discourses on political authorities in high-trust countries during the COVID-19 pandemic
Source: PLoS One. 2021 Jun 23;16(6):e0253175. doi: 10.1371/journal.pone.0253175 (PMC8221506; doi:10.1371/journal.pone.0253175)
Supplement: S5 Appendix — Includes results of the sentiment analysis including sample sizes before and after cleaning, average positive score (APS) with standard deviance, average negative score (ANS) with standard deviance, and sentiment tables (Figs) A-0 through -D3, which portray the distributions of tweets by sentiment score per country per phase. (PDF) [file pone.0253175.s005.pdf]

S5 Appendix: Sentiment analysis results

Base sets

Denmark

Sample size: 94,908 tweets

Sample size after cleaning: 94,288

Average positive score: 0.603 (sd=0.796)

Average negative score: -0.805 (sd=0.829)

	Pos				
Neg	0	1	2	3	4
0	25,20%	10,41%	5,03%	0,83%	0,04%
-1	22,16%	12,37%	5,25%	0,91%	0,03%
-2	7,47%	4,19%	1,77%	0,33%	0,01%
-3	1,99%	1,10%	0,52%	0,13%	0,00%
-4	0,13%	0,08%	0,04%	0,01%	

Figure A-0: Denmark, base set

Germany

Sample size: 100,000

Sample size after cleaning: 98,888

Avg. positive score: 0.385 (sd=0.649)

Avg. negative score: -0.604 (sd=0.939)

	Pos				
Neg	0	1	2	3	4
0	46,91%	13,63%	4,92%	0,29%	0,00%
-1	9,32%	3,35%	1,16%	0,07%	0,00%
-2	10,12%	3,39%	1,19%	0,07%	
-3	3,75%	1,18%	0,40%	0,07%	0,00%
-4	0,12%	0,04%	0,02%	0,00%	0,00%

Figure B-0: Germany, base set

The Netherlands

Sample size: 100,000

Sample size after cleaning: 99,161

Avg. pos. score: 0.424 (sd=0.722)

Avg. neg. score: -0.820 (sd=0.971)

	Pos				
Neg	0	1	2	3	4
0	35,95%	8,05%	4,51%	0,50%	0,01%
-1	19,28%	5,70%	3,09%	0,34%	0,01%
-2	9,71%	2,90%	1,69%	0,19%	0,01%
-3	5,09%	1,54%	0,93%	0,12%	0,00%
-4	0,23%	0,09%	0,04%	0,01%	0,00%

Figure C-0: The Netherlands, base set

Sweden

Sample size: 100,000 tweets

Sample size after cleaning: 99,197

Average positive score: 0.505 (sd=0.688)

Average negative score: -0.933 (sd=0.851)

	Pos				
Neg	0	1	2	3	4
0	22,30%	9,30%	2,35%	0,26%	0,01%
-1	25,18%	14,19%	3,62%	0,37%	0,02%
-2	9,85%	5,97%	1,71%	0,18%	0,00%
-3	2,16%	1,50%	0,50%	0,06%	0,01%
-4	0,22%	0,13%	0,10%	0,01%	

Figure D-0: Sweden, base set

Phase 1 (first case – March 10)

Denmark

Sample size: 321

Sample size after cleaning: 318

Avg. pos. score: 0.513 (sd=0.764)

Avg. neg. score: -0.997 (sd=0.811)

	Pos			
Neg	0	1	2	3
0	19,18%	6,60%	1,57%	0,63%
-1	29,87%	12,89%	5,35%	1,26%
-2	10,69%	3,77%	2,83%	0,31%
-3	3,46%	1,26%	0,31%	

Figure A1: Denmark, Phase 1

Germany

Sample size: 6125

Sample size after cleaning: 6061

Avg. pos. score: 0.347 (sd=0.622)

Avg. neg. score: -0.637 (sd=0.961)

	Pos			
Neg	0	1	2	3
0	48,80%	12,04%	4,41%	0,23%
-1	7,33%	2,38%	0,89%	0,07%
-2	12,80%	4,41%	1,45%	0,02%
-3	3,91%	0,87%	0,25%	0,02%
-4	0,10%		0,03%	

Figure B1: Germany, Phase 1

The Netherlands

Sample size: 3347

Sample size after cleaning: 3322

Avg. pos. score: 0.450 (sd=0.740)

Avg. neg. score: -0.879 (sd=0.947)

	Pos				
Neg	0	1	2	3	4
0	31,37%	7,62%	5,36%	0,27%	0,03%
-1	20,23%	5,60%	3,40%	0,36%	0,03%
-2	12,70%	3,94%	2,35%	0,15%	
-3	4,49%	1,02%	0,72%	0,15%	
-4	0,12%	0,06%	0,03%		

Figure C1: The Netherlands, Phase 1

Sweden

Sample size: 591 tweets

Sample size after cleaning: 586

Average positive score: 0.515 (sd=0.701)

Average negative score: -1.003 (sd=0.784)

	Pos				
Neg	0	1	2	3	4
0	14,16%	8,70%	2,22%	0,17%	
-1	32,08%	17,92%	3,58%	0,51%	0,17%
-2	9,73%	3,58%	2,05%	0,17%	
-3	3,07%	1,54%		0,17%	
-4			0,17%		

Figure D1: Sweden, Phase 1

Phase 2 (March 11 – March 25)

Denmark

Sample size: 2,492

Sample size after cleaning: 2,487

Avg. pos. score: 0.650 (sd=0.797)

Avg. neg. score: -0.914 (sd=0.814)

Neg \ Pos	0	1	2	3	4
0	19,14%	9,93%	3,90%	0,68%	0,08%
-1	22,60%	15,32%	5,79%	1,13%	0,08%
-2	9,33%	5,71%	2,53%	0,16%	0,04%
-3	1,57%	1,09%	0,52%	0,16%	
-4	0,04%	0,12%	0,08%		

Figure A2: Denmark, Phase 2

Germany

Sample size: 26,390

Sample size after cleaning: 26,205

Avg. pos. score: 0.375 (sd=0.651)

Avg. neg. score: -0.506 (sd=0.864)

Neg \ Pos	0	1	2	3	4
0	51,13%	13,81%	5,45%	0,30%	
-1	8,11%	2,40%	0,97%	0,09%	0,00%
-2	9,93%	3,01%	1,34%	0,06%	0,00%
-3	2,23%	0,76%	0,28%	0,02%	
-4	0,08%	0,01%	0,01%		

Figure B2: Germany, Phase 2

The Netherlands

Sample size: 25,950

Sample size after cleaning: 25,811

Avg. pos. score: 0.524 (sd=0.780)

Avg. neg. score: -0.874 (sd=0.958)

	Pos				
Neg	0	1	2	3	4
0	29,84%	8,85%	6,23%	0,68%	0,00%
-1	17,86%	6,55%	3,69%	0,41%	0,02%
-2	11,80%	4,37%	2,51%	0,34%	0,00%
-3	4,23%	1,47%	0,80%	0,09%	
-4	0,18%	0,05%	0,03%	0,00%	

Figure C2: The Netherlands, Phase 2

Sweden

Sample size: 2667 tweets

Sample size after cleaning: 2658

Average positive score: 0.552 (sd=0.712)

Average negative score: -0.964

(sd=0.820)

	Pos				
Neg	0	1	2	3	4
0	18,17%	9,56%	2,48%	0,26%	
-1	26,22%	15,69%	4,74%	0,45%	0,04%
-2	9,74%	6,43%	1,92%	0,26%	
-3	2,22%	0,87%	0,38%	0,08%	
-4	0,26%	0,15%	0,08%		

Figure D2: Sweden, Phase 2

One month after

Denmark

Sample size: 1,010

Sample size after cleaning: 1,005

Avg. pos. score: 0.618 (sd=0.782)

Avg. neg. score: -0.905 (sd=0.829)

Neg	0	1	2	3	4
0	19,50%	8,96%	5,47%	0,60%	0,10%
-1	23,88%	15,22%	5,07%	0,70%	
-2	9,35%	4,38%	2,09%	0,20%	
-3	2,29%	1,19%	0,70%	0,10%	
-4	0,10%		0,10%		

Figure A3: Denmark, Phase 3

Germany

Sample size: 13,716

Sample size after cleaning: 13,623

Avg. pos. score: 0.343 (sd=0.621)

Avg. neg. score: -0.542 (sd=0.882)

	Pos				
Neg	0	1	2	3	4
0	50,79%	12,93%	4,45%	0,24%	0,03%
-1	8,89%	2,66%	0,97%	0,06%	0,01%
-2	11,31%	3,09%	1,01%	0,05%	
-3	2,33%	0,76%	0,26%	0,01%	
-4	0,04%	0,01%	0,10%		

Figure B3: Germany, Phase 3

The Netherlands

Sample size: 9,456

Sample size after cleaning: 9,396

Avg. pos. score: 0.535 (sd=0.794)

Avg. neg. score: -1.022 (sd=1.007)

	Pos				
Neg	0	1	2	3	4
0	25,64%	7,87%	5,41%	0,60%	
-1	17,78%	6,44%	3,54%	0,66%	0,01%
-2	14,29%	4,87%	3,27%	0,39%	0,02%
-3	5,59%	1,92%	1,10%	0,15%	0,01%
-4	0,30%	0,07%	0,04%	0,03%	

Figure C3: The Netherlands, Phase 3

Sweden

Sample size: 1138 tweets

Sample size after cleaning: 1134

Average positive score: 0.599 (sd=0.756)

Average negative score: -1.047 (sd=0.799)

	Pos				
Neg	0	1	2	3	4
0	13,93%	7,58%	2,29%	0,71%	0,09%
-1	27,78%	18,08%	4,14%	0,97%	
-2	9,79%	7,23%	2,20%	0,62%	
-3	2,47%	1,50%	0,26%	0,09%	
-4	0,09%	0,18%			

Figure D3: Sweden, Phase 3
