# Supplementary material for: Lockdowns, lethality, and laissez-faire politics. Public discourses on political authorities in high-trust countries during the COVID-19 pandemic
Source: PLoS One. 2021 Jun 23;16(6):e0253175. doi: 10.1371/journal.pone.0253175 (PMC8221506; doi:10.1371/journal.pone.0253175)
Supplement: S6 Appendix — Includes Tables A1-D3 with the results of the topic modeling analysis. (PDF) [file pone.0253175.s006.pdf]

S6 Appendix: Topic modeling results

Abbreviations:

Gov't=government

Rtw=retweet

DK: Denmark

DE: Germany

NL: The Netherlands

SE: Sweden

TABLE A1: DENMARK, PHASE 1 (K=20, ITERATIONS=2000, WORDS-PER-TOPIC=20, N=321[1])			
	**Topic**	**Wgt**	**Topic words**
Topic 1	***COVID-19 in DK***	0.239	corona - dkpol - kan - regeringen - så - coronavirus - covid - regeringdk - regering - hvorfor - mere - godt - ved - dkmedier - hvordan
Topic 2	***Cancelling large events***	0.098	mette - frederiksen - coronavirus - arrangementer - deltagere - covid - aflyses - mere - bør - danmark - aflyse - statsminister - https://ift.tt - siger - dkpol
Topic 3	***Economic bailout***	0.040	regeringen - dkbiz - klar - regeringdk - covid - virksomheder - vigtigt - økonomisk - corona - dkøko - hjælpe - hjælp - erhvervslivet - danske - følger
Topic 4	***Political meeting + intensive care capacity***	0.037	covid - sundpol - dkpol - erhvervsliv - smittet - må - lønmodtagere - borger - få - danske - lave - sengepladser - folketinget - udviklingen - mødes
Topic 5	***Economic compensation to businesses + citizenship ceremonies***	0.031	give - hånd - politisk - dansk - regeringen - corona - kompensation - snakke - pga - åbner - håndtryk - p.g.a - virussen - statsborger - penge
Topic 6	***New containment measures***	0.030	statsministeren - nye - coronavirus - tiltag - gå - inddæmme - coronavirus-hvornår-ser-statsministeren-gå - undtagelsestilstand - talerstolen - hvornår - ser - https://www.information.dk/indland/leder - varsler - t.co&utm_content - btn&utm_source
Topic 7	***Italy: containment strategy + DK strategy incites panic***	0.029	coronavirus - regeringen - italien - fordi - mest - indtil - undgå - forkert - afhænger - stoppe - viser - regeringens - smittede - panik - rammes
Topic 8	***Criticism of prime minister***	0.028	heunicke - regeringdk - statsmin - dkpol - coronavirus - sundpol - dkmedier - sstsundhed - dksundhed - ramt - gåafmette - rigspoliti - ansvar - https://www.danmarksfremtid.dk/mette-frederiksen-coronavirus - gåafheunicke
Topic 9	***Gov't and politicians***	0.026	helt - får - dage - christiansborg - medier - enig - allerede - samme - ift - coronavirusdk - stoppet - bl.a - regering - flere - tysk
Topic 10	***Comparison with other epidemics***	0.023	samme - så - corona - influenza - virus - fugleinfluenza - forbindelse - siden - finde - efterfølgende - kina - steder - overfor - tiltag - bare
Topic 11	***Travel to Italy***	0.023	myndighederne - får - fly - få - rejse - norditalienere - italiens - fordi - døve - styr - sikkerhed - sted - mon - info - alternativet
Topic 12	***Italy: schools***	0.022	skoler - regeringen - lukke - coronavirus - grund - italien - universiteter - landet - italienske - lukket - rss - verden - udbruddet - seneste - italiens
Topic 13	***Vulnerable citizens***	0.022	bare - regeringen - dø - udsatte - koster - medierne - borgere - alligevel - ihjel - influenza - ældre - reagerer - tro - indsats - alm
Topic 14	***Press conference***	0.022	direktøren - ved - statsministeren - corona-virus - sstsundhed - covid - mindst - pressemøde - blod - adgang - erhvervsstyrelsen - udenrigsministeriet - borgerservice - organisation - rigspolitichefen
Topic 15	***Political opportunism***	0.022	mette - frederiksen - score - point - næsten - forholdsregler - mindske - selvom - stor - tager - hvorfor - utroligt - artikler - seriøst - score-nemme-point.html
Topic 16	***Expert: more political transparency needed (psychology) (retweet)***	0.019	regeringdk - bør - viser - vores - smitte - lykkes - html - kommunikationstrategi - ændre - bag - psykologien - forsker - forskning - https://threadreaderapp.com/thread - m_b_petersen
Topic 17	***Children in refugee camps***	0.018	regering - mellemøsten - sammen - mens - sidder - børn - uge - tidligere - kommer - uden - kampe - https://m.economictimes.com/news/international/world-news/in-coronavirus-fight-china-gives-citizens-a-color-code-with-red-flags/articleshow - bus - lov - afgør
Topic 18	***Containment strategy criticism***	0.017	lige - håndtere - dine - vide - iwar - statsborgere - absurd - dennis - forstår - par - økonomisk - sundhedsstyrelsen - udlandet - statsoverhovedet - danmarks
Topic 19	***Citizenship ceremonies cancelled***	0.013	håndtryk - stop - stedet - uden - regeringdk - forklaring - term - forurening - flygtninge - statsborgerskab - hænder - før - krav - politikerne - demonstration
Topic 20	***Expert: more political transparency needed (psychology) (rtw. Article)***	0.012	begynder - spille - kort - åbne - lukke - helt - regeringen - bne-kort - ndrer-kurs - regeringen-%c - corona-krisen-kan-lukke-samfundet-helt - https://politiken.dk/debat/kroniken/art - kurs - ændrer - corona-krisen

[1] Please note that the cleaned sample size differs slightly from sentiment analysis results, since a few results were excluded from the sentiment analysis due to program error, while some results had to be excluded from the topic modeling analysis, if the same person had written the same things repeatedly in order to avoid entire topics coming out as the result of a single tweet.

Table A2: Denmark, Phase 2 (K=20, iterations=2000, words-per-topic=20, n=2460)			
	**Topic**	**Wgt**	**Topic words**
Topic 1	***COVID-19 and the gov't***	0.336	covid - så - regeringen - dkpol - corona - kan - regeringdk - coronadk - må - ved - regering - coronavirus - dkmedier - danmark - hvorfor -
Topic 2	***Thank yous + solidarity***	0.133	covid - regeringdk - tak - regeringen - dkpol - corona - sammen - statsministeren - kan - hinanden - stor - vores - godt - andre - tage -
Topic 3	***Praise for prime minister***	0.116	mette - frederiksen - covid - dkpol - statsminister - coronadk - vores - pressemøde - respekt - lige - coronavirusdk - tak - dkmedier - tale - godt -
Topic 4	***Bailout***	0.085	dkpol - covid - regeringen - regeringdk - virksomheder - dkbiz - kan - selvstændige - hjælp - dansk - hjælpepakke - hjælpe - danske - små - nye -
Topic 5	***Gov't failed***	0.082	dkmedier - dkpol - covid - regeringdk - eudk - statsmin - sstsundhed - fejlet - heunicke - https://www.danmarksfremtid.dk/coronavirus-danske-medier - svigtet - ansvar - https://www.danmarksfremtid.dk - sstbrostrom - ledelse -
Topic 6	***Frederiksen: do social distancing, do not panic buy***	0.052	statsministeren - covid - hamstre - må - coronavirusdk - handle - danskerne - hold - danskere - folk - afstand - hamstring - dronningen - sagde - mennesker -
Topic 7	***Lockdown***	0.050	covid - lukker - coronavirus - regeringen - danmark - statsministeren - grænser - lukket - hjem - siger - skoler - dag - morgen - danske - april -
Topic 8	***New containment measures***	0.048	mette - frederiksen - coronavirus - statsminister - danmark - pressemøde - aften - covid - nye - onsdag - tiltag - dk/samfund - corona - personer - kommer -
Topic 9	***Press conference***	0.037	dkpol - covid - mere - regeringdk - statsministeren - marts - læs - http://coronasmitte.dk - https://www.regeringen.dk/nyheder/pressemøde - spejlsalen - regeringen - personer - undskyld - pressemøde - danmark -
Topic 10	***Tripartite agreement***	0.031	regeringen - coronavirus - parter - arbejdsmarkedets - trepartsaftale - vidtgående - job - regering - model - folketinget - lønmodtagere - giver - miste - danske - lovforslag -
Topic 11	***Strategy: COVID-19 vs. climate crisis***	0.029	corona - regeringen - regering - ved - tilbage - dkgreen - regningen - penge - hjælp - klima - uden - skat - klimakrisen - folketing - pension -
Topic 12	***Risks of different containment strategies***	0.028	regering - usa - svenske - sverige - folkestyret - spiller - coronakrisen - forsøger - frygt - lars - så - kina - nationen - præsident - trump -
Topic 13	***Call for ban on large events***	0.028	regering - flere - covid - forsamlinger - kræver - liste - dødsfald - fået - flertal - hvilke - oplysninger - sygdom - gennem - forbud - corona-virus -
Topic 14	***Prime minister YouTube video***	0.027	unge - statsministeren - spørgsmål - vigtigt - byx-mxz - corona - youtube - https://youtu.be/p - rasmus - paludan - svarer - hver - ide - demokrati - egne -
Topic 15	***Compulsory admission***	0.026	dkpol - regeringdk - dkmedier - covid - coronavirus - uden - adgang - hjem - venstredk - private - jakobbellemann - coronavirusdk - sanktioner - lade - enhedslisten -
Topic 16	***COVID-19 in developing countries***	0.026	kan - millioner - coronavirus - regeringen - smitter - dø - så - kun - virus - corona-støtte - behandling - giver - mio - mennesker - smitte -
Topic 17	***Criticism of gov't communication, leadership***	0.024	covid - regeringdk - dkbiz - hamstring - statsmin - dkmedier - dkpol - https://www.danmarksfremtid.dk/coronavirus-danmark-hamstring - ledelse - kommunikation - sundpol - dårlig - nød - aldrig - ellers -
Topic 18	***Single Twitter user***	0.024	corona - regeringen - nye - artikler - svar - artikel - millionerenudfordring-hercules.blogspot.com - https://den - tidligt - berlingske - dkbiz - danmark - jydske - politiken - vestkysten -
Topic 19	***Gov't is deceptive (rtw: article)***	0.018	corona - danskerne - test - regeringen - lyset - bag - mere - taler - teststrategi - offensiv - f%c - avis - korte - brug - skrevet -
Topic 20	***Asylum seekers DK vs. DE***	0.018	corona - smittet - asylansøgere - grund - tyskland - danske - asylstop - regering - marts - intet - gør - smittefaren - grænsen - avis - korte -

Table A3: Denmark, Phase 3 (K=20, iterations=2000, words-per-topic=20, n=1001)			
	**Topic**	**Wgt**	**Topic words**
Topic 1	***Reopening***	0.601	covid - dkpol - regeringen - kan - corona - så - regeringdk - danmark - coronadk - statsministeren - mere - godt - må - ved - åbne -
Topic 2	***Press conference***	0.109	mette - frederiksen - coronavirus - danmark - mette-frederiksen - berlingske - coronakrisen - går - jydske - pressemøde - ssi - sundhed - altinget - vestkysten - derfor -
Topic 3	***Containment strategy and the elderly***	0.096	corona - regeringen - plan - ved - år - hvem - stille - ældre - dør - test - lige - gør - fordi - taler - spørgsmål -
Topic 4	***Media as propaganda channels***	0.090	dkmedier - dkpol - covid - regeringdk - eudk - statsmin - https://www.edtwodthnb.dk - sstsundhed - propaganda - newsdk - mikrofonholderi - spin - dag - kommunikation - presselogen -
Topic 5	***Schools reopening: COVID-19-infected homes***	0.054	børn - regeringen - skole - hjemmet - møde - forældre - vigtigt - tak - presset - daginstitutioner - besluttet - børnene - skolen - starte - smitte -
Topic 6	***Healthcare sector reopening***	0.040	regeringen - sundhedsvæsenet - aften - antallet - smittede - vores - danske - kommunerne - aftalt - lille - presse - aftale - velfærdslov - flertal - regionerne -
Topic 7	***Strategy for vulnerable citizens***	0.038	regeringen - sårbare - udsatte - stærkt - rette - initiativer - venstre - lederskab - særligt - finde - hjælp - sundheds - risiko - dksocial - kommende -
Topic 8	***Bailout for sports clubs***	0.038	store - joymogensen - støtte - handler - gennem - pga - dybt - danske - personer - hjælp - kulturmin - besluttet - september - forsamlinger - sætter -
Topic 9	***Business bailout expansion***	0.035	covid - regeringen - dkpol - dkbiz - partier - folketingets - https://www.regeringen.dk/nyheder - enige - mere - justere - direktør - regeringdk - erhverv - dansk-økonomi - indgået -
Topic 10	***No faith in gov't, gov't power hungry***	0.034	statsministeren - tillid - politisk - magt - skridt - danskerne - sagt - frygt - kritisk - ansvar - leder - reglerne - talt - sst - overfor -
Topic 11	***Citizenship ceremonies without handshakes***	0.034	regeringen - håndtryk - grundlovsceremonier - mattiastesfaye - udlændinge - midlertidigt - suspendere - nye - integrationsministeriet - nemlig - gør-danskerne - hold-afstand-lød-rådet-så - håndtrykket - spolitik - suspendere-handtryk -
Topic 12	***International strategies***	0.031	coronavirus - regering - italien - kære - landets - respekt - spanien - restauranter - masker - arbejdsskade - stor - britiske - caféer - frankrig - upassende -
Topic 13	***Criticism from Lars Løkke***	0.031	løkke - lars - egen - sendt - situation - fanget - information - bør - føre - vacciner - https://politiken.dk/indland/art - debat - lige - rasmussen - egne -
Topic 14	***Gov't and prime minister show leadership***	0.031	venstre - radikale - regeringen - venstredk - dansk - står - strategien - sfpolitik - enhedslisten - spolitik - lyder - opposition - hånd - mandater - suk -
Topic 15	***Swedish strategy***	0.030	usa - forskere - sammen - sektor - bruger - sverige - klima - lader - svenske - sej - slå - må - private - følge - mens -
Topic 16	***Single Twitter user***	0.028	index.htm - spørgsmål/s - https://www.ft.dk/samling - tro - følger - faktisk - hvordan - sundpol - sundhedssystemet - udviklingen - påstår - erhvervsminister - ministeren - stiller - bruges -
Topic 17	***Raeson.dk articles***	0.027	regering - coronakrisen - løn - https://www.raeson.dk - coronakrise - coronadk - gratis - kommentar - glad - solidaritet - zealands - dkpol - raesonekstra - abir - procent -
Topic 18	***New Zealand gov't takes paycut***	0.025	vild - iwar - anbefaling - forskellige - folkets - tæt - slår - præcis - opfordring - udløser - folket - næsten - https - simpelt - svenskerne -
Topic 19	***Poland bans abortion***	0.025	økonomisk - gøre - flygtninge - mht - polen - danskindustri - tiltag - påfaldende - tragedie - medlemmer - netop - corona-krisen - forhindre - abort - heldigvis -
Topic 20	***Children back in school, comply with proper behavior***	0.024	statsminister - giver - april - virus - børnenes - børnene - indvandrere - avis - jeres - stikken - imponeret - genåbnet - vildt - forklaring - skole -

TABLE B1: GERMANY, PHASE 1 (K=20, ITERATIONS=2000, WORDS-PER-TOPIC=20, N=6124)			
	**Topic**	**Wgt**	**Topic words**
Topic 1	***COVID-19 in DE***	0.305	regierung - coronavirus - corona - covid - deutschland - virus - mehr - schon - mal - china - menschen - deutsche - gibt - merkel - immer -
Topic 2	***Turkey breaks migration pact***	0.156	merkel - corona - coronavirus - frau - deutschland - kanzlerin - cdu - covid - türkei - flüchtlinge - eigentlich - coronavirusde - mal - grenzen - schon -
Topic 3	***The Minister of Health***	0.061	covid - merkel - coronavirus - spahn - coronavirusde - jensspahn - deutschland - gesundheitsminister - coronavirusdeutschland - regierung - die_regierung - jens - coronadeutschland - bundesregierung - sarscov -
Topic 4	***COVID-19 in Northern Italy***	0.047	regierung - coronavirus - italien - html - zahl - millionen - menschen - quarantäne - norden - betroffen - wegen - lombardei - covid - italienische - gebiete -
Topic 5	***Italy: containment measures***	0.035	regierung - coronavirus - schulen - italien - wegen - covid - abgesagt - märz - veranstaltungen - html - maßnahmen - geschlossen - großveranstaltungen - ausbreitung - frankreich -
Topic 6	***COVID-19 in China***	0.034	regierung - coronavirus - china - html - wuhan - chinesische - peking - infizierte - chinesischen - chinas - folgen - virus - wut - schweigen - erster -
Topic 7	***Seehofer denies handshake with Merkel***	0.032	merkel - coronavirus - seehofer - angela - hand - corona - verweigert - grund - cdu - bundeskanzlerin - https://www - kanzlerin - berlin - wegen - handschlag -
Topic 8	***Italy: lockdown***	0.031	regierung - coronavirus - italien - italienische - wegen - städte - abriegeln - ausbreitung - land - maßnahmen - ganze - infektionen - drastischen - html - zwei -
Topic 9	***COVID-19 internationally***	0.030	regierung - coronavirus - covid - iran - neue - warum - polen - chinesische - gibt - mktcid - youtube - corona - gefühl - iranische - afjflf -
Topic 10	***Merkel incompetent***	0.027	regierung - coronavirus - merkel - deutschland - tun - china - angesichts - inkompetent - wahrscheinlich - fall - weiterhin - erst - schauen - denke - notstand -
Topic 11	***Lack of communication from chancellor***	0.027	merkel - corona - frau - kanzlerin - corona-virus - angela - twttr.shr - coronavirus - schaffen - bild.html - krise - sprechen - vertrauen - warum - bild.html?wtmc -
Topic 12	***Virologist: containment measures necessary***	0.026	regierung - coronavirus - covid - youtube - maßnahmen - vorsorge - coronavirus-ausbreitung - japanische - berichtet - virologe - infiziert - beruhigt - japan - chinesischen - html -
Topic 13	***Global fatalities***	0.022	coronavirus - regierung - menschen - macht - millionen - sterben - weltweit - html - kranke - umgang - alte - journalismus - hl_diff - alten - ältere -
Topic 14	***Call for Chancellor to take the lead***	0.021	coronavirus - regierung - Twitter#echobox - chinesische - chefsache - kampf - autofeed&utm_medium - social&utm_source - utm_term - umgang - fdp - kritisiert - fpö - folgen - utm_source -
Topic 15	***China: strategy and economy***	0.020	maß - mitte - merkel - coronavirus - welt - vorgehen - socialmedia.Twitter.shared.web - hongkong - regierung - wirtschaft - https://www.welt.de/vermischtes/article - legt - umgang - fest - https://www.welt.de/wirtschaft/article -
Topic 16	***Gov't overwhelmed****	0.019	regierung - coronavirus - steht - veröffentlicht - chinas - tonline_news - html - afrika - versagt - führung - kämpft - trauen - faznet - krise - zeigt -
Topic 17	***COVID-19 in the US***	0.019	virus - trump - status - corona - kampf - kontrolle - sogar - regierung - https://Twitter.com/isabelvillalon - merkel's - geht - usa - steckt - donald - scheiße -
Topic 18	***Conspiracy: gov't secret plan***	0.017	regierung - corona - entdeckt - geheimplan - ausbruch - corona-geheimplan - covid - corona-folgen - regierung-entdeckt - https://www.wallstreet-online.de/nachricht - corona-geheimplan-regierung-entdeckt - lange - prüft - konjunkturprogramm - ende -
Topic 19	***Conspiracy: NWO***	0.016	merkel - wuhan - hält - coronavirus-ausbruch - nwo-rede - kurz - coronavirus - nmry - youtube - https://youtu.be/chyndj - itb - html - ungarn - erstmals - iran -
Topic 20	***Climate change and migration***	0.016	deutsche - reisewarnung - wegen - lebensmittel - coronavirus - forschungsministerin - klimawandel - südtirols - heute - nwo - bringen - corona-krise - sollen - migrationspakt - hamburg -

Table B2: Germany, Phase 2 (K=20, iterations=2000, words-per-topic=20, n=26107)			
	**Topic**	**Wgt**	**Topic words**
Topic 1	***COVID-19 in DE***	0.154	corona - regierung - merkel - covid - menschen - coronavirus - mehr - mal - schon - leute - frau - immer - warum - coronavirusde - bitte -
Topic 2	***Call for political action***	0.137	merkel - covid - regierung - corona - coronavirus - deutschland - maßnahmen - coronadeutschland - spahn - schon - coronavirusde - geht - wäre - handeln - kanzlerin -
Topic 3	***Merkel holds speech***	0.117	merkel - covid - coronavirus - coronavirusdeutschland - corona - bundeskanzlerin - frau - kanzlerin - coronavirusde - ansprache - angela - rede - coronakrise - deutschland - coronadeutschland -
Topic 4	***Gov't vs. AfD***	0.094	corona - merkel - regierung - coronavirus - afd - schon - seit - virus - mal - mehr - covid - krise - wer - frau - deutschland -
Topic 5	***Borders stay open for asylum seekers***	0.055	merkel - grenzen - deutschland - coronavirus - covid - corona - schließen - schulen - coronavirusde - dicht - mehr - regierung - wegen - grenze - flüchtlinge -
Topic 6	***Curfew***	0.048	regierung - coronavirus - maßnahmen - covid - wegen - ausbreitung - ausgangssperre - kampf - bleiben - geschlossen - heute - mehr - corona - spanien - weitere -
Topic 7	***COVID-19 internationally***	0.044	regierung - coronavirus - covid - china - deutschland - corona - italien - merkel - virus - zahl - fälle - mehr - millionen - wuhan - html -
Topic 8	***Press conference: new containment measures***	0.044	merkel - coronavirus - bundeskanzlerin - pressekonferenz - maßnahmen - länder - angela - kanzlerin - corona - covid - live - uhr - ministerpräsidenten - corona-krise - heute -
Topic 9	***Business bailout***	0.035	regierung - coronavirus - corona-krise - corona - wirtschaft - html - unternehmen - plant - euro - milliarden - kredite - hilfspaket - covid - folgen - wegen -
Topic 10	***Merkel exposed to COVID-19***	0.034	merkel - quarantäne - coronavirus - kontakt - kanzlerin - angela - bundeskanzlerin - arzt - getestet - positiv - corona-infiziertem - wegen - negativ - html - wurde -
Topic 11	***Merkel: "Es ist ernst"***	0.033	merkel - corona-krise - angela - bundeskanzlerin - ernst - html - coronavirus - fernsehansprache - kanzlerin - Twitter - ansprache - nehmen - deutsche - abend - hält
Topic 12	***Tim Kellner videos (alt right activist)***	0.029	merkel - coronavirus - angela - youtube - kontakte - sozialkontakte - covid - corona - corona-krise - soziale - verzichten - tim - kellner - möglich - https://youtu.be -
Topic 13	***Political parties***	0.028	merkel - cdu - covid - spd - corona - coronavirus - csu - spahn - deutschland - coronavirusde - fdp - afd - grüne - söder - coronavirusdeutschland -
Topic 14	***Merkel: 60-70% of all Germans will be infected***	0.024	merkel - coronavirus - deutschland - prozent - welt - corona-krise - corona - regierung - html - infizieren - Twitter#echobox - angela - social&utm_source - krise - tun -
Topic 15	***Protection of the population***	0.023	regierung - corona - covid - wegen - coronavirus - html - krise - mehr - demokratie - schützen - welt - müssen - merkel - besonders - corona-krise -
Topic 16	***No communication from the gov't (rtw: article)***	0.021	kanzlerin - merkel - twttr.shr - corona - coronavirus - corona-krise - rede - bild.html - führung - bild.html?wtmc - auftritt - regierung - bild - krise - kommentar -
Topic 17	***Merkel: COVID-19 is an immense challenge***	0.021	merkel - solidarität - corona-krise - seit - coronavirus - herausforderung - weltkrieg - größte - probe - kanzlerin - zweiten - corona - covid - html - hilferuf -
Topic 18	***COVID-19 and Donald Trump***	0.018	regierung - trump - coronavirus - usa - corona - merkel - bla - deutsche - donald - versucht - impfstoff - welt - curevac - covid - sichern -
Topic 19	***Crisis support to the cultural sector (rtw)***	0.013	coronavirus - regierung - merkel - kultur - bureg - covid - reagieren - unterstützung - kulturstaatsministerin - grütters - https://www.bundesregierung.de/bregde/-bundesregierung/staatsministerin - verspricht - notlagen - künstlern - härtefälle-reagieren -
Topic 20	***Gov't and federal Länder make agreement***	0.009	bundesregierung - deutschland - regierungschefs - corona-epidemie - regierungschefinnen - merkel - bureg - angesichts - bundesländer - bundesländer-angesichts - vereinbarung https://www.bundesregierung.de-/bregde/aktuelles/vereinbarung - https://www.bundesregierung.de/breg-de/themen/buerokratieabbau/vereinbarung - https://www.bundesregier-ung.de/breg-de/themen/coronavirus/besprechung - besprechung -

Table B3: Germany, Phase 3 (K=20, iterations=2000, words-per-topic=20, n=7268)			
	**Topic**	**Wgt**	**Topic words**
Topic 1	***COVID-19 in DE***	0.201	corona - regierung - merkel - covid - mehr - schon - coronavirus - immer - menschen - deutschland - gibt - mal - kommt - seit - viele -
Topic 2	***COVID-19 in Nordrhein-Westfalen***	0.139	merkel - corona - covid - mal - frau - regierung - laschet - gut - coronavirusde - macht - kanzlerin - coronavirus - wer - afd - heute -
Topic 3	***Merkel makes statement***	0.067	merkel - angela - regierungserklärung - bundeskanzlerin - kanzlerin - corona-krise - coronavirus - bundestag - covid - forsch - lange - pandemie - kritisiert - corona - lockerungen -
Topic 4	***Öffnungsdiskussionsorgien (opening discussion orgies) (Merkel statement)***	0.051	merkel - öffnungsdiskussionsorgien - html - coronavirus - lockerungen - kanzlerin - kritisiert - warnt - kritik - angela - corona-lockerungen - corona - corona-maßnahmen - länder - bundeskanzlerin -
Topic 5	***Compulsory facemasks in NRW***	0.050	covid - merkel - deutschland - coronavirus - coronavirusde - corona - coronakrise - coronavirusdeutschland - lockdown - frau - html - politik - maskenpflicht - raus - cdu -
Topic 6	***Austrian gov't takes paycut***	0.038	regierung - corona - cdu - spd - corona-krise - csu - wegen - covid - kurz - geht - maßnahmen - deutschland - grünen - österreich - deutsche -
Topic 7	***Reopening***	0.037	regierung - covid - coronavirus - maßnahmen - corona - april - mai - ende - krise - lockerung - corona-maßnahmen - wer - https://bit.ly - wochen - fordert -
Topic 8	***COVID-19 origin***	0.037	coronavirus - regierung - merkel - wuhan - sekunde - china - sicherheit - corona-krise - wiegen - html - ausbruch - trump - covid - welt - warum -
Topic 9	***Compulsory facemasks***	0.035	regierung - maskenpflicht - covid - masken - corona - coronavirus - spahn - maske - merkel - html - tragen - virus - krise - gesundheitsministerium - coronadeutschland -
Topic 10	***Gov't line vs. expert recommendations***	0.028	merkel - corona - prof - covid - youtube - kanzlerin - lockdown - corona-krise - neue - https://www.youtube.com/watch?v - rki - rki-zahlen - widersprechen - gates - homburg -
Topic 11	***Corona bonds***	0.027	merkel - corona - angela - europa - corona-krise - politik - corona-bonds - italien - deutschland - geht - kontrolle - bundeskanzlerin - macron - solidarität - bereit -
Topic 12	***Conspiracy: Merkel and China created COVID-19***	0.023	coronavirus - merkel - schützt - labor - nrw - chinesischem - https://youtu.be - youtube - mgz - qxbi - laschet - regierung - öffnen - schulen - armin -
Topic 13	***Öffnungsdiskussionsorgien (opening discussion orgies) (Merkel statement)***	0.023	merkel - welt - corona-lockerungen - https://www.welt.de/politik/-deutschland/article - streit - kanzlerin - kritisiert - ton - bundesländer - socialmedia.Twitter.shared.web - öffnungsdiskussionsorgien - vergreift - lockerungen - kanzlerin-vergreift - wegen -
Topic 14	***Criticism of gov't by Jens Lehmann***	0.022	regierung - merkel - frau - covid - geld - corona-politik - jens - mehr - kritisiert - bravo - lehmann - genre - betreff - youtube - corona-pandemie -
Topic 15	***Criticism of gov't by Sciencefiles.org***	0.022	regierung - corona - corona-krise - merkel - macht - html - kippt - folgen - shutdown - energiewende - probleme - corona-heiligsprechung - neue - https://www.t-online.de/nachrichten/deutschland/id - schließt -
Topic 16	***Gov't rhetoric of fear (rtw: article)***	0.020	regierung - angst - html - merkel - corona-starre - deutschen - politik - Twitter - focus-kolumne - utm_source - news - html?rnd - versetzt - live-ticker - bürger -
Topic 17	***Virologist: reopening is the wrong signal***	0.017	lockerungen - welle - regierung - gesendet - zweite - signal - falsches - cdefc - falsches-signal-gesendet-a-af - https://www.spiegel.de/wissenschaft/-medizin/corona-zweite-welle-regierung - covid - derspiegel - corona - spiegel - updayde -
Topic 18	***Online petition for climate friendly financial aid***	0.017	merkel - angela - stellen - zukunft - menschen - Twitter - bundeskanzlerin - fordere - zentrum - greenrecovery - corona-rettungspläne - online-petition - wwf_deutschland - gruenekonjunktur&utm_source - share&utm_campaign -
Topic 19	***Linke: give financial aids to illegals***	0.017	menschen - merkel - euro - fordern - seehofer - linke - corona-hilfe - papiere - corona - regierung - dad - fc-a - papiere-a-f - euro-hilfe - https://-www.spiegel.de/politik/deutschland/corona-virus-linken-politiker-fordern -
Topic 20	***Chancellor power hungry (rtw)***	0.016	rande - kubicki - kanzlerin - merkels - corona-politik - bewegt - amtsanmassung - cbdb - amtsanmassung-a-fdfabd - kanzlerin-bewegt - https://www.spiegel.de/politik/deutschland/wolgang-kubicki - grundrechte - derspiegel - regelungskompetenzen - masst -

Table C1: The Netherlands, Phase 1 (K=20, iterations=2000, words-per-topic=20, n=3321)			
	**Topic**	**Wgt**	**Topic words**
Topic 1	***Call for political action***	0.300	minpres - rivm - coronavirus - covid - rutte - nederland - corona - coronavirusnederland - maatregelen - mensen - coronavirusnl - italie - regering - gaan - gaat
Topic 2	***Hand shaking***	0.156	rutte - handen - coronavirus - schudden - coronanederland - persconferentie - corona - hand - covid - minpres - rivm - nederland - handenschudden - premier - mark
Topic 3	***Patient care, the elderly***	0.115	corona - regering - mensen - coronavirus - virus - gaan - ouderen - griep - rutte - dood - patienten - gaat - goed - laten - hele
Topic 4	***Turkey breaks migration pact***	0.075	corona - rutte - coronavirus - virus - grenzen - minpres - vvd - dicht - gaat - europa - land - turkije - open - crisis - krijgen
Topic 5	***COVID-19 in Brabant***	0.062	coronavirus - thuis - evenementen - covid - mogelijk - werken - brabant - maatregelen - blijven - indamfase - coronanederland - scholen - grote - gaan
Topic 6	***Infectivity, lethality of COVID-19***	0.041	corona - mensen - getest - week - testen - ziekenhuis - patient - test - besmet - blok - ligt - quarantaine - kom - lang - thuis
Topic 7	***Gov't prioritizes economy***	0.034	rutte - corona - telegraaf - Twitter - referral&utm_campaign - t.co&utm_medium - https://www.telegraaf.nl/t - stootje - corona-patienten - verkeerde - stelt - https://www.telegraaf.nl/nieuws - prioriteit - kwijt - nederland
Topic 8	***Forming a gov't (Belgium)***	0.034	regering - corona - coronavirus - komt - zaken - land - federale - crisis - virus - vormen - duidelijk - politici - belgische - nodig - situatie
Topic 9	***Italy: schools***	0.034	regering - coronavirus - italiaanse - maatregelen - italie - bedrijven - scholen - covid - maand - getroffen - maart - corona - sportevenementen - federale - publiek
Topic 10	***Infected, dead of COVID-19 in NL***	0.030	corona - doden - griep - overleden - coronavirus - man - virus - besmet - mensen - Twitter&utm_medium - iran - social&utm_campaign - socialsharing_web - blijft - kans
Topic 11	***COVID-19 strategies internationally***	0.030	corona - regering - wereld - media - virus - trump - nie - grootste - chinezen - hysterie - politiek - miljard - china - ter - natuurlijk
Topic 12	***Rejection of early COVID-19 debate***	0.030	regering - coronavirus - rutte - corona - gevolgen - recht - baudet - debat - minister - uitbraak - hoog - politieke - aanpak - pakken - land
Topic 13	***European cooperation***	0.029	coronavirus - rutte - europese - samenwerking - nos - wenselijk - gaat - https://nos.nl/l - economie - gebruikt - staat - vanzelf - minister-president - liegen - klap
Topic 14	***Emergency scenarios***	0.029	coronavirus - rutte - kabinet - aanpak - werkt - deskundigen - noodscenario's - bepalen - noodscenarios - rutte-kabinet-werkt - nl/politiek - https://www - coronavirus.html - nl-nl/bb - http://a.msn.com
Topic 15	***Containment strategy***	0.029	rutte - coronavirus - minister - crisisoverleg - volgende - voorbereiding - morgen - nodig - corona-fase - nos - premier - ministers - bruins - fase - persconferentie
Topic 16	***News outlets***	0.027	minpres - nos - rivm - coronavirusnederland - bruno_bruins - minvws - persconferentie - telegraaf - kijk - jinek - coronavirusnl - rtlnieuws - corona - npo - nieuwsuur
Topic 17	***Air travel***	0.027	corona - minpres - ziek - goed - wachten - lekker - rutte - tweets - zien - tonen - schiphol - media - mooie - wist - hulp
Topic 18	***Statistics and knowledge – who to trust***	0.025	regering - den - haag - covid - risico - verhaal - experts - groei - cijfers - artsen - inclusief - status - deskundige - corona-uitbraak - kop
Topic 19	***Extra containment measures***	0.024	rutte - stop - handen - schudden - extra - kondigt - coronavirus - corona-maatregel - nl/coronavirus - https://www - handen-schudden.html - rutte-kondigt-extra-corona-maatregel - gaan - oproep - maatregelen
Topic 20	***Italy: lockdown***	0.022	quarantaine - regering - coronavirus - italie - inwoners - sluit - https://www - kwart - coronavirus.html - nl/buitenland - noorden - italiaanse - regering-italie-sluit-noorden - nunl - miljoen

TABLE C2: THE NETHERLANDS, PHASE 2 (K=20, ITERATIONS=2000, WORDS-PER-TOPIC=20, N=21842)			
	**Topic**	**Wgt**	**Topic words**
Topic 1	***COVID-19 strategy***	0.228	covid - minpres - rivm - nederland - coronavirusnederland - rutte - coronavirus - lockdown - coronavirusnl - coronanederland - maatregelen - gaat - gaan - mensen - coronacrisis
Topic 2	***Minister President Rutte***	0.158	rutte - corona - allemaal - zegt - mensen - coronavirus - krijgen - virus - minpres - echt - gaat - mark - covid - coronanederland - gaan
Topic 3	***Effectiveness of herd immunity***	0.149	rutte - corona - mensen - virus - groepsimmuniteit - coronavirus - covid - coronanederland - laten - gaan - gaat - krijgen - doden - bevolking - ouderen
Topic 4	***Press conference***	0.144	rutte - minpres - coronanederland - toespraak - covid - coronavirus - goed - coronacrisis - corona - duidelijk - speech - mark - elkaar - goede - premier
Topic 5	***Gov't prioritizes economy***	0.121	corona - rutte - regering - coronavirus - crisis - gaat - vvd - economie - land - gaan - virus - geld - wereld - kabinet - laat
Topic 6	***Gov't line vs. expert recommendations***	0.117	rutte - rivm - corona - coronavirus - regering - kabinet - wilders - laat - baudet - aanpak - coronavirusnederland - covid - experts - coronadebat - deskundigen
Topic 7	***Closing chools***	0.108	scholen - kinderen - rutte - minpres - coronavirus - dicht - open - corona - covid - coronavirusnederland - sluiten - blijven - school - thuis - ouders
Topic 8	***Infectivity statistics***	0.074	corona - doden - mensen - aantal - nederland - minpres - besmettingen - rivm - patienten - coronavirus - covid - ziekenhuizen - rutte - week - ziekenhuis
Topic 9	***Economy***	0.062	zorg - minpres - corona - regering - mensen - coronavirus - geld - coronacrisis - bedrijven - rutte - covid - betalen - onderwijs - kabinet - extra
Topic 10	***Extra containment measures***	0.059	regering - maatregelen - coronavirus - nieuwe - verspreiding - maart - corona - covid - gaan - genomen - nederland - april - sluiten - nederlandse - neemt
Topic 11	***Press conference***	0.058	rutte - coronavirus - premier - land - spreekt - persconferentie - corona - mark - uur - nos - morgen - toespraak - live - maandag - maatregelen
Topic 12	***Lockdown***	0.056	corona - minpres - thuis - mensen - coronavirus - rutte - werken - werk - regering - mogelijk - blijf - blijven - jaar - huis – dag
Topic 13	***Social distancing***	0.053	mensen - afstand - minpres - houden - meter - coronacrisis - corona - elkaar - rutte - covid - coronavirusnl - regering - coronavirusnederland - persconferentie - regels
Topic 14	***Dutch politicians***	0.046	minpres - geertwilderspvv - thierrybaudet - rivm - vvd - jesseklaver - covid - coronavirus - bruno_bruins - ekamertweets - nos - robjetten - hugodejonge - coronavirusnederland – minvws
Topic 15	***Gov't decisions (Belgium)***	0.046	regering - corona - coronavirus - crisis - maatregelen - vertrouwen - federale - partijen - zaken - volmachten - zetten - vormen - nieuwe - strijd – covid
Topic 16	***Shortage of intensive care resources***	0.032	coronavirus - testen - mondkapjes - regering - covid - test - minpres - corona - china - vaccin - tekort - trump - nederland - chinese – testkits
Topic 17	***Rutte: a majority of Dutch people will be infected***	0.029	rutte - coronavirus - hamsteren - groot - besmet - deel - raken - nederlanders - https://www - bevolking - nl/coronavirus - virus - premier - covid – nodig
Topic 18	***Telegraaf articles***	0.021	coronavirus - Twitter - referral&utm_campaign - telegraaf - t.co&utm_medium - https://www.telegraaf.nl/nieuws - groepsimmuniteit - nederland - https://www.telegraaf.nl/t - jaap - mark - corona - straks – retorische
Topic 19	***No proof of herd immunity***	0.015	rutte - covid - risico - groot - https://www.dagelijksestandaard.nl - nek - arts - coronavirus - neemt - volkskrant - corona - regering - wetenschappelijke - groepsimmuniteit – lekker
Topic 20	***Greek refugee camps***	0.012	rutte - social&utm_campaign - Twitter&utm_medium - corona - socialsharing_web - utm_source - oproep - vluchtelingen - griekse - uitbraak - haal - vrees - eilanden - cbaae - uitbraak-corona

Table C3: The Netherlands, Phase 3 (K=20, iterations=2000, words-per-topic=20, n=9355)			
	**Topic**	**Wgt**	**Topic words**
Topic 1	***COVID-19 in NL***	0.533	corona - rutte - regering - mensen - minpres - gaan - gaat - covid - coronavirus - goed - virus - laat - economie - maatregelen - komen -
Topic 2	***Containment measures***	0.171	rutte - persconferentie - covid - minpres - coronavirus - coronamaatregelen - coronacrisis - corona - vragen - coronavirusnederland - mark - vraag - nos - premier - coronavirusnl -
Topic 3	***Parliament, politicians***	0.167	rutte - corona - crisis - vvd - kabinet - zorg - gaat - coronacrisis - land - minpres - mark - beleid - regering - media - staat -
Topic 4	***Containment measures***	0.157	minpres - covid - rivm - hugodejonge - coronavirus - mondkapjes - coronavirusnederland - coronacrisis - coronavirusnl - coronanederland - nederland - coronamaatregelen - coronadebat - rutte - vvd -
Topic 5	***Retirement homes: infection increase***	0.107	corona - rutte - ouderen - zorg - minpres - coronavirus - mensen - mondkapjes - testen - rivm - verpleeghuizen - personeel - besmet - hugodejonge - beschermen -
Topic 6	***Validity of RIVM statistics***	0.103	corona - doden - rivm - aantal - mensen - rutte - cijfers - nederland - week - miljoen - lockdown - hoeveel - getest - virus - landen -
Topic 7	***Facemasks sent to China in February***	0.087	rutte - trump - corona - coronavirus - china - rivm - baudet - februari - januari - regering - nederland - kabinet - strijd - laat - begin -
Topic 8	***Reopening schools***	0.074	kinderen - minpres - open - scholen - rivm - covid - coronavirus - meter - coronavirusnederland - rutte - afstand - persconferentie - school - ouders - gaan -
Topic 9	***Bailouts***	0.064	geld - minpres - coronacrisis - rutte - corona - miljard - klm - betalen - euro - eurobonds - wbhoekstra - regering - steun - miljarden - bedrijven -
Topic 10	***Lockdown***	0.049	coronavirus - rutte - maatregelen - persconferentie - april - premier - mei - corona - regering - gaan - evenementen - verlengd - mark - vanavond - komt -
Topic 11	***Bill Gates conspiracy***	0.044	nieuwe - rutte - normaal - corona - coronavirus - samenleving - middel - mark - nwo - hoax - new - gates - bill - trump - news -
Topic 12	***Containment measures (Belgium)***	0.039	regering - covid - corona - boodschap - leven - hoop - exit - corona-lockdown - peiling - belgische - federale - natie - sophie_wilmes - hoeder - mondmaskers -
Topic 13	***Privacy: corona-apps***	0.036	app - corona - rutte - nodig - verplicht - regering - corona-apps - economie - apps - dreun - coronaapp - privacy - mark - kamer - corona-app -
Topic 14	***Bill Gates conspiracy***	0.035	corona - telegraaf - Twitter - t.co&utm_medium - referral&utm_campaign - rutte - https://www.telegraaf.nl/nieuws - https://www.telegraaf.nl/t - gates - vaccin - werkt - bill - who - medicijn - zaken -
Topic 15	***Rutte admits mistakes***	0.032	rutte - gemaakt - fouten - corona-aanpak - ten - https://www - gezondheid - vrijheid - ongetwijfeld - koste - nl/coronavirus - nunl - ongetwijfeld-fouten-gemaakt.html - gaan - nederland -
Topic 16	***British investigation into China***	0.029	regering - coronavirus - covid - china - onderzoek - britse - wereld - wuhan - chinese - vriendjes - ter - amerikaanse - iwar - massaal - voorkomen -
Topic 17	***Retirement homes: Rutte's mother's home***	0.027	social&utm_campaign - Twitter&utm_medium - socialsharing_web - rutte - verpleeghuis - den - utm_source - coronavirus - corona-diktatuur - mark - haag - moeder - adc - ruttes-moeder - vandaag -
Topic 18	***Facemasks***	0.027	regering - covid - mondkapjes - merkel - mondkapje - macron - maskers - slechte - youtube - duitse - dragen - zie - rutte&cs - openbaar - super -
Topic 19	***Rutte advises caution***	0.027	the - regering - spijt - achteraf - covid - voorzichtigheid - beter - artikel - https://bit.ly - team - and - jan - lees - brief - open -
Topic 20	***Negative expert feedback (psychology)***	0.015	groningen - zelden - rutte - domme - psycholoog - opstand - corona-aanpak - gehoord - maatregelen - komt - martin - Twitter - appelo - corona-aanpak-rutte - komt-opstand

TABLE D1: SWEDEN, PHASE 1 (K=20, ITERATIONS=2000, WORDS-PER-TOPIC=20, N=587)			
	**Topic**	**Wgt**	**Topic words**
Topic 1	***COVID-19 in SE***	0.949	corona - regeringen - regering - sverige - ska - kommer - bara - får - covid - viruset - vill - statsminister - svpol - coronavirus - svenska -
Topic 2	***Gov't performance***	0.392	coronavirus - covid - svpol - swedishpm - löfven - stefan - iran - coronaviruset - folkhalsomynd - corona - fall - socialdemokrat - italien - åtgärder - karantän -
Topic 3	***Borders stay open for asylum seekers***	0.075	komma - såg - flyktingar - säga - bakom - står - fattar - panik - utanför - flyr - skall - kontroller - https://bladet.se/nyheter/flyktingar-fran-syrien-bar-pa-coronavirus-regeringen-godtar-frivilliga-kontroller - naiva - grekisk -
Topic 4	***Crisis council***	0.071	media - mindre - krishanteringsrådet - epg/regeringen-kallar-in-myndigheter - kallas - senfärdighet - aldrig - kanske - statsminister - krisrad - https://www.aftonbladet.se/nyheter/samhalle/a/ew - riket - alltså - regioner - ygeman -
Topic 5	***Gov't incompetence***	0.067	mest - eftersom - inkompetenta - skolor - måste - fram - italien - region - kunna - visar - korrupt - avbryter - ang - löser - kalla -
Topic 6	***Preparedness***	0.062	beredskap - god - vården - bryr - riksdagen - situationen - informeras - medierna - onsdagen - finlands - behandlade - lugn - sju - värsta - regeringens -
Topic 7	***Prime minister holds meeting***	0.067	expressen - löfven - stefan - twtr - Twitter&social - social_sharing&utm_source - link&utm_campaign - ställer - möte - grund - eget-mote - https://www.expressen.se/nyheter/stefan-lofven-kallar - kallar - sydafrika - resa -
Topic 8	***Welfare***	0.055	tidigare - äldre - första - problemet - magdalena - lite - bra - vecka - landstinget - rad - välfärden - beröm - andersson - finansdepsv - https://ledarsidorna.se -
Topic 9	***Crisis council***	0.053	myndigheter - kallar - epg - https://www.aftonbladet.se/a/ew - krisråd - företag - namn - sr_ekot - people - löner - utsatta - pressträff - sänka - stämmer - massa -
Topic 10	***Call for political action***	0.052	följer - minut - utvecklingen - noga - följa - hurra - stund - chansen - oerhört - lösning - års - krafttag - flyktingvåg - kommit - mkt -
Topic 11	***COVID-19 in the US***	0.050	nästan - betala - usa - handlar - smittan - höjd - tänka - hotet - ena - testet - långt - sämsta - https://www.aftonbladet.se/a/qldnem - samtal - extrapersonal -
Topic 12	***Traveling and high risk zones***	0.047	sade - statsministern - sydafrika - noll - resa - gjorde - utbrott - leder - precis - ramx/virusprofessorn-varsta-epidemin - riskområde - listar - storbritannien - vips - lista -
Topic 13	***Italy: sports events***	0.047	corona-viruset - italienska - även - grund - serie - publik - lugna - gått - sänka - corona-utbrottet - också - samt - skyller - gör - regeringen -
Topic 14	***Criticism of gov't***	0.047	nya - socialdemokraterna - krismöte - klarar - sprider - skit - fått - vfoypn - http://bit.ly - suicide - depression - prata - beslut - triumfer - gigantiska -
Topic 15	***Israel's strategy (positive response)***	0.046	sätter - israel - melodifestivalen - redan - sjukdom - hälsofarlig - början - väl - först - medborgare - besked - medier - sociala - res - syns -
Topic 16	***Infectivity***	0.046	åtgärder - spridningen - nödvändiga - vidtagit - samhället - hittills - trygg - spara - bongo - pensionsutbetalningar - välkommet - vidta - sjukdom - andra-lander-stanger-granser - varnar -
Topic 17	***Strategy: COVID-19 vs. refugee crisis***	0.046	hand - svenskar - nytt - hjälp - test - trots - hellre - livet - föredöme - kommuner - arbetslöshet - fråga - tält - låta - kritiken -
Topic 18	***Political opportunism***	0.044	tills - läs - igår - fantastiskt - dödar - bort - släpper - möjligheten - tanke - nära - påstår - upprörande - coronavirusdebatten-demokraternas-nya-bluff - hyllar - tyskl -
Topic 19	***Spread prevention***	0.043	vilja - sittande - stora - testas - därför - store - påverkar - tänker - brukar - suck - syndare - församlingen - munskydd - systemet - berörda -
Topic 20	***Armed Forces: secret COVID-19 plan***	0.040	osv - ansvaret - mette - publik - corona-beredskap - januari - planerar - svenska - tänk - frederiksen - högerextrema - misströnde - hemlig-corona-beredskap - försvaret - hemlig

TABLE D2: SWEDEN. PHASE 2 (K=20, ITERATIONS=2000, WORDS-PER-TOPIC=20, N=2594)			
	Topic	Wgt	Topic words
Topic 1	*COVID-19 in SE*	0.606	corona - regeringen - regering - kommer - ska - covid - bara - får - sverige - måste - andra - mer - göra - få - finns -
Topic 2	*Swedish authorities*	0.504	covid - regeringen - sverige - svpol - swedishpm - coronavirussverige - coronavirus - corona - coronasverige - folkhalsomynd - gör - coronaviruset - regering - skolor - tegnell -
Topic 3	*Prime minister holds speech*	0.147	löfven - stefan - statsminister - tal - coronavirussverige - corona - statsministern - svpol - nationen - covid - svt - håller - presskonferens - coronasverige - bra -
Topic 4	*Business bailout*	0.086	regeringen - företag - corona - covid - miljarder - coronasverige - bra - svpol - kommer - staten - extra - mer - åtgärder - krispaket - anledning -
Topic 5	*Gov't and Folkhälsomyndigheten*	0.079	beslut - tegnell - vill - komma - regeringen - anders - corona-viruset - fatta - tiden - såg - fick - folkhälsomyndigheten - spelar - fattar - hos -
Topic 6	*Ban on large gatherings*	0.057	fler - beslutat - personer - regeringen - sammankomster - coronavirus - beslutet - mars - offentliga - förbud - danska - statsminister - centerpartiet - tillfälligt - allmänna -
Topic 7	*High schools and universities closing*	0.057	hemma - regeringen - skolan - stanna - barn - skolorna - coronasverige - elever - studenter - uppmanar - stänga - covid - föräldrar - stängs - beslut -
Topic 8	*Online petition: quarantine Sweden proactively*	0.049	löfven - stefan - karantän - sätt - sverige - stoppa - proaktivt - corona-spridningen - change - petition - artikel - sign - https://sverigesradio.se/sida/artikel.aspx?programid - sr_ekot - sydkorea -
Topic 9	*Healthcare capacity, esp. protective gear*	0.047	slut - covid - socialstyrelsen - skyddsutrustning - lenahallengren - sjukhus - https://ledarsidorna.se - första - världens - sjukvård - utrustning - ansvariga - personal - enbart - uppdrag -
Topic 10	*Single Twitter user*	0.041	löfven - stefan - sverige - öppna - gränser - dag - iran - kriminella - varenda - virus - landet - invandrare - ryssland - smittad - charterresor -
Topic 11	*Jimmie Åkesson (SD) travels to Greek refugee camps*	0.039	åkesson - statsminister - jimmie - väl - kristersson - förra - politiska - fått - journalister - ulf - senaste - samtal - veckan - turkiet - sprängningar -
Topic 12	*Criticism of gov't*	0.039	politik - visar - svpol - politiker - ger - samhälle - beredskap - inse - världen - regering - dinröst - starkt - fhm - dags - hävdade -
Topic 13	*Refugees from high-risk zones*	0.034	hit - världen - delar - drabbade - åka - fortsätter - hårt - migranter - ifrån - värst - inreseförbudet - afterski - hem - problem - undantas -
Topic 14	*New restrictions (Finland)*	0.033	finland - finlands - restauranger - social - marinsanna - förbereder - öppet - https://svenska.yle.fi/artikel - följ - restriktioner - rekommendationer - införa - statsminister - coronavirus - Twitter-share&utm_medium -
Topic 15	*Criticism of gov't*	0.033	samt - skriver - långt - ännu - vänstern - ifrån - gång - ministrar - sätt - fokus - agerat - någonting - framöver - arbetet - kört -
Topic 16	*Single Twitter user*	0.031	svtnyheter - regionstockholm - del:tw - äldre - stockholm - https://www.tv - region - svpol - personer - redo - coronasmittade - fruktansvärt - jö - play.se/program/nyhetsmorgon - ännu -
Topic 17	*Gov't vs. expert recommendations*	0.031	regeringens - pandemi - sjöfart - gå - milj - talet - lämnas - iwar - miljon - tillbaka - dog - handlingskraft - utifrån - verkar - hong -
Topic 18	*COVID-19 internationally*	0.030	trump - usa - coronavirus - kinesiska - tyska - betala - viruset - ser - köpa - kör - lyssnade - kinas - medlemmar - män - usas -
Topic 19	*Morgan Johansson as backup for Löfven*	0.027	expressen - sjuk - corona - löfven - twtr - Twitter&social - social_sharing&utm_source - link&utm_campaign - stefan - morgan - tar - johansson - partiledarna - corona/?utm_medium - lofven -
Topic 20	*Löfven's nationalistic behavior (rtw: video)*	0.026	youtube - nationalist - spåren - arga - bern - lars - februari - m-m - posts - redan - hansson - tragiskt - opposition - https://youtu.be/hrn - snälla -

Table D3: Sweden. Phase 3 (K=20, iterations=2000, words-per-topic=20, n=1120)			
	**Topic**	**Wgt**	**Topic words**
Topic 1	***COVID-19 in SE***	0.969	regeringen - corona - covid - sverige - regering - svpol - löfven - ska - kommer - stefan - coronasverige - många - får - bara - mer -
Topic 2	***Political parties***	0.156	covid - swedishpm - svpol - folkhalsomynd - sverige - socialdemokrat - coronavirussverige - lenahallengren - coronasverige - coronavirus - socialstyrelsen - strategi - tack - miljopartiet - migpol -
Topic 3	***Business bailout***	0.058	regeringen - ska - miljarder - företag - nya - coronasverige - stödet - miljoner - skriver - vabba - personalen - anledning - gälla - krispaket - dag -
Topic 4	***Care homes not sufficiently protected***	0.053	dör - gamla - munskydd - hårt - äldreboenden - resurser - arbetar - tester - extra - vanlig - drabbas - flockimunitet - drabbade - sjukhus - skydd -
Topic 5	***New Zealand gov't takes paycut***	0.052	nya - löner - regering - lön - zeelands - president - ekonomin - bort - unga - corona- sänker - imorgon - samhälle - egen - trump - myndighet -
Topic 6	***Call for political action, political transparency***	0.047	svt - trump - svtnyheter - statens - kritiserar - svaga - läser - olika - gjorde - statsminister - agerande - intressant - flesta - tystnad - medier -
Topic 7	***"Sweden for the Swedes" (mostly single Twitter user)***	0.047	sverige - dag - viruset - afghaner - svenskarna - islamister - diktatur - gränser - invandrare - landet - nyanlända - iran - analfabeter - bidrags - fortsätter -
Topic 8	***New restrictions on gambling***	0.047	åtgärder - minska - ekonomiska - regeringen - utbrottet - spelmarknaden - covid - följer - företagare - krav - företagen - krävs - tillfälligt - europas - vidtar -
Topic 9	***Abolishment of karensavdrag (self-paid first sick days)***	0.046	corona - bl.a - åkesson - tänka - gäller - dagens - jimmie - karensavdraget - tid - makten - problemet - positivt - nyheter - ang - räcker -
Topic 10	***Fatality: SE vs. rest of Fennoscandia***	0.045	sverige - myndigheterna - corona-pandemin-sverige-regeringen - dodligheten - norge - danmark - corona-pandemin - finland - myndigheterna-togs-pa-sangen - se/debatt-artiklar - http://www.news - sängen - togs - dödligheten - igen -
Topic 11	***Failed gov't***	0.044	misslyckats - läs - maste-politikerna-gripa-in - https://www.dn.se/debatt/folkhalsomyndigheten - agera - styr - dags - leder - knappast - beredskapslagren - hem - journalist - faktiskt - lyssna - covid -
Topic 12	***Löfven (Finland)***	0.043	statsminister - löfven - stefan - https://svenska.yle.fi/artikel - kom - jubla - läget - johan - person - analys - coronavirus - sade - öppnas - prata - bort -
Topic 13	***High mortality in SE****	0.041	fallet - lyckas - drar - riksdagen - viruset - ganska - lägga - motsvarar - införa - bekräftade - skillnaden - högt - flyg - sociala - utsatta -
Topic 14	***Lack of support for health care personel***	0.039	vårdpersonal - sviker - karensdagen - analyser - höll - anställda - minst - coronakrisen - svenska - tyvärr - slopa - försök - ansiktsmasker - ensamkommande - skatt -
Topic 15	***Sending children to school***	0.039	skolan - hemma - galla - två - människor - procent - barn - lärare - smittar - anna_ekstrom - spills - fredag - innebär - uteserveringar - föräldrar -
Topic 16	***No proof of herd immunity***	0.037	expressen - immunitet - bevis - aftonbladet - möjligt - säkra - tag - bort - förslag - testning - fem - förstår - html - antagande - osäkra -
Topic 17	***Expert calls Tegnell a fraud***	0.036	svenska - regeringen - forskare - jan - läkare - tegnell - kritisk - hanterat - stillström - corona-epidemin - virolog - pensionerad - tillsatt - docent - vården -
Topic 18	***Fatalities in care homes***	0.035	grupper - uppdrag - särskilt - äldreboenden - ersättning - forskning - vaccin - stoppa - peka-finger-at-varandra - ratt-tid - aldreboendena - https://www.dn.se/nyheter/sverige/lofven - http://dn.se - drabbas - insatserna -
Topic 19	***Gov't prioritizes economy***	0.035	pengar - tala - passar - kris - trodde - https://Twitter.com/believereuro/status - ordförande - vänstern - extra - svarar - mänskliga - regeringen-bara-loser-naringslivets-kris - https://www.dn.se/debatt/forvanande - miljarder - nedskärningar -
Topic 20	***Prime minister admits mistake***	0.030	svtnyheter - del:tw - fick - beräkningar - pappa - bromsen - betala - uppger - msbse - beredskapen - korrupta - betalas - information - kommer - tillrackligt-bra:nyh:lp -
