# Supplementary material for: Lockdowns, lethality, and laissez-faire politics. Public discourses on political authorities in high-trust countries during the COVID-19 pandemic
Source: PLoS One. 2021 Jun 23;16(6):e0253175. doi: 10.1371/journal.pone.0253175 (PMC8221506; doi:10.1371/journal.pone.0253175)
Supplement: S1 File — (DOCX) [file pone.0253175.s007.docx]

For **S1, S5** and **S6 Appendices**, the following naming scheme for tables and figures applies:

|  | **Base sets (0)** | **Phase 1 (1)** | **Phase 2 (2)** | **Phase 3 (3)** |
| --- | --- | --- | --- | --- |
| **Denmark (A)** | A-0 | A1 | A2 | A3 |
| **Germany (B)** | B-0 | B1 | B2 | B3 |
| **The Netherlands (C)** | C-0 | C1 | C2 | C3 |
| **Sweden (D)** | D-0 | D1 | D2 | D3 |
