# Supplementary material for: Lockdowns, lethality, and laissez-faire politics. Public discourses on political authorities in high-trust countries during the COVID-19 pandemic
Source: PLoS One. 2021 Jun 23;16(6):e0253175. doi: 10.1371/journal.pone.0253175 (PMC8221506; doi:10.1371/journal.pone.0253175)
Supplement: Attachment — Submitted filename: Response to Reviewers_april2021.docx [file pone.0253175.s008.docx]

# Response to Reviewers

First of all we want to thank the editor for the opportunity to revise the manuscript. We would like to thank the reviewers and the editor for their insightful and constructive feedback. This has helped us to improve the paper considerably.

Please find the responses to the points raised by the editor and reviewers below.

Editor

1.Style requirements

RESPONSE: We have made sure the manuscript follows the style requirements.

2.Ethics

RESPONSE: We have added an ethical statement in the methodology section.

3.Include captions

RESPONSE: We have included captions for our supporting information.

Reviewer #1

*Commendable effort in conducting the study and putting this manuscript together. Perhaps, it could be better if the section on 'Data and Method' (i.e. line 224) and the section on 'Data' (i.e. line 232) could be combined as 'Data Collection' and placed as sub-sections under the section on 'Methodology' (i.e. line 266). Moreover, the section title 'Results and Discussion' should be changed to 'Results' as there is already a section on 'Discussion' later on. Please also revisit the References and try to be consistent about the style of referencing. It is an interesting study. Well tried.*

RESPONSE: We want to thank the reviewer for the compliments and the useful feedback.

We agree with the suggested changes in the structure and have adjusted the manuscript accordingly.

Structure in the Data and Methods section changed to:

Methodology

- Data collection
- Method of analysis

Results and Discussion section changed to “Results” and the “Discussion” subsection changed to a main section.

Reviewer #2

*General - Needs a review for grammar/ punctuation.*

RESPONSE: We have reviewed the manuscript on this and improved grammar/punctuation.

*Introduction - The first paragraph merits many more citations - currently, there are none.*

RESPONSE: We agree with the reviewer that more references are warranted. We have added multiple references, both with regards to COVID-19 and to the issue of trust.

*Introduction - I think there is an important difference between trust in government and trust in authorities - the two ideas are discussed interchangeably here, but I think they merit teasing out if possible. For example, in the US there is a difference between mistrust in the CDC/ FDA and mistrust in the Trump/ Biden administrations. They’re connected, but also separate. Someone can have low trust in a political administration but high trust in a health authority.*

RESPONSE: We agree with this comment. Our paper focuses on trust in governments or political trust, not trust in health authorities. We have clarified this. We have added “political” when we mention authorities throughout the article to ensure there is no misunderstanding.

RESPONSES TO SPECIFIC COMMENTS PER LINE:

Line 36: RESPONSE: changed “some time” to “a month”. Dates are added.

Line 45: RESPONSE: we corrected dangling parenthesis.

Line 61: RESPONSE: we added citations.

*Line 65: Personally, I don’t see the need for an entire “literature review” section, unless requested by the journal. I think much of this woudl be better placed in an introduction, before you state the aims of the paper because it sets the paper up to flow nicer. After I read the introduction, I had a few questions that appear to be answered in the literature review section. I also think the literature review section can be condensed quite a bit, with some of the sentences that aren’t specifically relevant (like the part on ebola in West/Central Africa) just included as a citation, and not given so much prominence. This will make it easier to merge the literature review section into the introduction.*

RESPONSE: We see the point of the reviewer that the current literature review is strictly speaking not a literature review and might therefore be confusing. At the same time, we think it is relevant to provide theoretical background and it appeared difficult to fully include this in the introduction. We have followed the example of Wicke & Bolognesi (2020), which is cited, and changed the section name to “Theoretical background”. We hope this is acceptable for the reviewer. Furthermore, we followed the advice of the reviewer to take out certain parts (incl. parts on ebola) which were not specifically relevant.

Line 91: RESPONSE: we changed ‘skeptic’ to ‘skeptical’.

*Line 114: This seems too drastic, and the sentence is a bit confusing following the previous one*

RESPONSE: we changed “is bound to” to “may”. If we understand the reviewer correctly, they felt like the statement that “the population may even be more forgiving of an exaggerated response than an insufficient one” was contradicted by the statement that “too much confidence may actually be harmful in the long run”. Since response refers to actions and confidence to attitude (the word rhetoric is mentioned in the subsequent section as well), the intent was to signal that these statements refer to different aspects of the crisis response. In order to highlight the difference, we changed the word “response” in the former statement to “strategy”. If we misunderstood the comment, we are, of course, open to change this.

Line 160: RESPONSE: we added more references.

Line 172: RESPONSE: We have shortened this section a bit, although we do want to highlight a couple of substantial characteristics of the contexts in various countries.

*Line 177: I dont think that you’ve established how you determined that there is a high level of trust in each of these countries. Is this based on a citation? A hunch? I’d like to see more information about how you determined trust in each of the 4 countries, given the topic of the paper*.

RESPONSE: We base ourselves on background literature. The reviewer is correct that more references are needed to support this statement. In the new version the description of trust has changed:

“Statistical data consistently shows these four countries to have a high level of population trust in their authorities (OECD, 2020; Eurobarometer, 2015; Mungiu-Pippi et al., 2015; Our World in Data, 2018; The World Bank, 2020); as such, these four countries provide a valuable case study of the effects of different containment strategies on public opinion in high-trust countries.”

Line 225: RESPONSE: We added dates by bringing Appendix C into the main text (table 1). We provide an argumentation for these dates.

Line 232: RESPONSE: We have added a more detailed description of GetOldTweets, incl. sources on the software as well other researchers using it.

Line 253: RESPONSE: Thank you for the observation. Indeed, it may not have been clear that we were referring specifically to political authorities throughout the paper. We have corrected this throughout, and it should be clear now.

Line 262: REPONSE: We have added more details on geographical allocation.

Line 263: RESPONSE: We think the additional details should cover this point but let us know in case more information is needed.

Line 276: RESPONSE: “Marshmellows are healthy” is changed to “Merkel is a competent leader”.

Line 283: RESPONSE: Apologies, the appendices were not correctly named. We adjusted this.

Line 284: RESPONSE: Details on no-sentiment tweets have been added. In short: they are included and simply have a value of 0 when calculating the avg sentiment.

Line 291: RESPONSE: Some more info on topic modelling on short texts have been added. Some extra references and a little more detail at the beginning of the “Method of analysis” section have been added.

Line 483: RESPONSE: We agree that the conclusions at times express too much certainty. We have rewritten the conclusion. “it is clear” has been changed to “it seems to be the case”.

Line 502: RESPONSE: We have corrected this; ‘of’ added after regardless

Line 504: RESPONSE: We have added sources.

Line 513: RESPONSE: We agree and have changed “playing fast and loose with citizens’ lives” to “treating citizens’ lives with recklessness”.

Line 517: RESPONSE: We changed ‘may be less an expression’ to ‘is less an expression’

Line 567: RESPONSE: We added a citation.

Line 584: RESPONSE: We agree and have the conclusion on conspiracy theories removed as not really central to the main thesis of our article.

Appendices moved around according to the notes of R2.

Appendix B 🡪 A

Appendix D 🡪 B

Appendix A 🡪 C

Appendix H 🡪 D

Appendix G 🡪 E

Appendix with TM results 🡪 F (indeed, this was missing from the uploaded data – our apologies)
