# Supplementary material for: Lockdowns, lethality, and laissez-faire politics. Public discourses on political authorities in high-trust countries during the COVID-19 pandemic
Source: PLoS One. 2021 Jun 23;16(6):e0253175. doi: 10.1371/journal.pone.0253175 (PMC8221506; doi:10.1371/journal.pone.0253175)
Supplement: Attachment — Submitted filename: Response to Reviewers_May2021.docx [file pone.0253175.s009.docx]

# Response to Reviewers, second revision

We are delighted that our revisions in round 1 have met with approval and that only small adjustments are considered needed in this stage. Once again, we would like to thank the editor for the opportunity to revise our manuscript and the reviewer for their insightful suggestion.

Please find the response to the point raised by the reviewer below.

Reviewer #1:

*The authors have adequately addressed comments from the previous round of review of this manuscript.
It would even be better if the authors could briefly introduce the study design and also highlight ethical considerations about the study (this may be inserted in line 235). Other than that, this is a good paper to publish*

RESPONSE: We agree that an introduction to the study design at the beginning of the ‘Methodology’ section would improve the coherence of that section. We have thus added an overview of the research design under line 235 as suggested by the reviewer, which also includes ethical considerations about the study.
